# Relationships between Psychopathology, Psychological Process Variables, and Sociodemographic Variables and Comparison of Quarantined and Non-Quarantined Groups of Malaysian University Students in the COVID-19 Pandemic

**DOI:** 10.3390/ijerph18189656

**Published:** 2021-09-14

**Authors:** Nicholas Tze Ping Pang, Sandi James, Nelbon Giloi, Syed Sharizman Syed Abdul Rahim, Azizan Omar, Mohammad Saffree Jeffree, Firdaus Hayati, Mei Ching Lim, Mohd Amiruddin Mohd Kassim, Jun Rong Ng

**Affiliations:** 1 Faculty of Medicine and Health Sciences, University Malaysia Sabah, Kota Kinabalu 88400, Sabah, Malaysia; nicholas@ums.edu.my (N.T.P.P.); sandi.james@latrobe.edu.au (S.J.); nelbon.giloi@ums.edu.my (N.G.); syedsharizman@ums.edu.my (S.S.S.A.R.); azizan.omar@ums.edu.my (A.O.); saffree@ums.edu.my (M.S.J.); m_firdaus@ums.edu.my (F.H.); jennmclim@gmail.com (M.C.L.); ryanng.95.jr@gmail.com (J.R.N.); 2 Department of Social Work and Social Policy, School of Science, Health and Engineering, La Trobe University, Bundoora, Melbourne, VIC 3086, Australia

**Keywords:** anxiety, COVID-19, depression, psychological mindedness, stress

## Abstract

The COVID-19 pandemic has had considerable psychological health impacts across the globe. This study aimed to establish the psychological process variables underlying psychopathology in Malaysian public university students during the national Movement Control Order (MCO). The aim was to craft structured and sustainable psychological support programs with these students. We conducted a cross-sectional study involving Malaysian university students subjected to the Malaysian MCO. Structured questionnaires measuring sociodemographic factors, measures of depression, anxiety, stress, psychological mindedness, psychological flexibility and state mindfulness were employed. A total of 515 students participated in this study with 12 students (2.3%) being quarantined at the time. Many of them scored ‘moderate’ or above on the Depression, Anxiety and Stress Scale (DASS) with 20.2%, 25.0% and 14.2%, respectively. Quarantined students had higher depressive symptoms, with female students scoring significantly higher for depression, anxiety, and stress. Multiple regressions suggested gender and quarantine status predicted depression scores. However, only gender significantly predicted anxiety and stress. Psychological flexibility and psychological mindedness (Insight subscale) are significantly correlated with depression, anxiety, and stress, with psychological mindedness predicting all three psychopathologies. This study demonstrates that gender, psychological flexibility, and psychological mindedness are key demographic and psychological factors impacting students. Targeting psychological flexibility and psychological mindedness may enable timely prevention and intervention programs for our students to support their mental and physical health as we move through, and out of, the pandemic.

## 1. Introduction

The COVID-19 pandemic has had unprecedented and untold effects across world. In Malaysia, due to an increasing number of cases, a Movement Control Order (MCO) was implemented on 18 March 2020 and has repeatedly been extended. The MCO, while necessary, appeared to be generating an increasing level of psychological distress. While evidence of levels of psychological distress within the general population of Malaysia is currently still unavailable, a nationwide survey in China revealed that at least one-third of participants reported psychological distress [1].

With the high prevalence of psychological distress, this pandemic not only highlighted the importance of tailoring public health measures to physical health, but also stressed the impact of a pandemic on mental health. As such, undoubtedly psychiatrists and psychologists need to play a paramount role in the current world crisis, on different levels. At the general public level, they need to manage both the short and long term consequences of COVID-19 for mental health, while at the healthcare workers (HCWs) level, they have to support them psychologically, as HCWs are at risk of having distress from increased workload and constant fear of COVID-19 exposure [2]. Mental health issues are also addressed by the World Health Organization (WHO) through the integration of mental health and psychosocial support (MHPSS) into the COVID-19 response effort, re-establishing the necessity for the involvement of psychiatrists and psychologists in this pandemic [3].

University undergraduates are at a higher risk than the general population for this psychological distress to progress to a diagnosable psychiatric disorder. Leaving home, transitioning to adulthood, seeking employment, and becoming financially independent alongside large student debt increases this risk [4,5]. In Malaysia, on 18 March 2020, a large proportion of undergraduates were abruptly trapped on campus as the MCO was announced overnight [6,7]. These students were ordered to remain in their respective campuses and campus dormitories for months to prevent massive interstate movement across the country. The majority of learning processes were interrupted as educational institutions struggled to convert to online learning environments. Students were left, with little support, to ruminate about the status of their studies and their future, alongside the increasing COVID-19 cases being reported worldwide. They were expected to adhere to strict Standard Operating Procedures (SOP), such as social distancing, mandatory usage of face masks and frequent hand hygiene. Social gatherings on campus were not allowed, and students were told to remain in their own dormitory or hostel until further notice. Studies conducted prior to COVID-19 had demonstrated a higher prevalence of stress in this same group of undergraduates [8]. This suggests an urgent need to establish factors associated with increased psychopathology in students. This would enable mental health and psychological support services to be proactive amongst at-risk groups and to make these services readily available and accessible. It would assist in the development of tailored interventions.

Examining underlying psychological process variables, such as psychological mindedness, mindfulness, and psychological flexibility, can assist in the development of intervention initiatives. These variables have been chosen as they are reported as factors that may contribute to psychopathology in previous studies in a similar university population [9]. Psychological Mindedness (PM) is defined as an awareness of one’s inner psychological processes and attitude towards these processes [10]. PM has been found in previous studies to have inverse correlations with depressive symptoms and other indices of psychopathology [11,12]. State mindfulness is defined as increased attention and awareness in everyday life, and has also been shown to have inverse correlations with psychopathology [13,14]. Psychological Flexibility (PF) is the ability to experience the present moment actively and with awareness, changing or persisting with behavior as a function of contextual contingencies and valued goals [15]. There are robust relationships between a low level of PF and psychopathology, with higher levels of PF contributing to increased psychological wellness [16]. All three psychological process variables are related to third wave mindfulness-based therapies [17], and numerous studies have shown that they are strongly correlated [18,19]. However, they do measure distinctly separate qualities. PM is derived from psychodynamic and psychoanalytic traditions [20], while PF is a construct that measures treatment outcomes in Acceptance and Commitment Therapy (ACT) [21]. Psychological mindedness and psychological flexibility are important in the ability to ‘drop the struggle’ with one’s own thoughts, and the ability to ‘unhook’ from distressing thoughts [22,23]. On the other hand, high dispositional mindfulness enhances well-being and helps in dealing with stressful situations such as the COVID-19 pandemic [24].

These relationships are especially present in quarantined groups who are forced into lockdown due to compulsory MCO and Enhanced MCO (EMCO) laws. There are many studies that document the psychological harms of segregation, including associations between solitary confinement and self-harm, anxiety, depression, paranoia, and aggression, among other symptoms [25]. Various studies pertaining to solitary confinement in incarceration demonstrate mental health issues are more likely to occur. Symptoms of psychological distress was reported in around half the participants with one-fifth demonstrating severe psychopathology [26,27]. In the intervening year since COVID-19 established a foothold, there have been multiple studies in similar groups that demonstrate psychological distress during enforced quarantines and movement restriction [28,29,30]. These symptoms were shown to persist or increase over time. It then becomes crucial to examine sociodemographic and psychological process variables that maintain or reduce health during quarantine (essentially solitary confinement under national legislation without the stigma of incarceration). Evidence from this study combined with previous literature can help inform how some people may respond to extended MCO as well as lengthy periods of quarantine and isolation.

The aims of this study are as follows:

- to ascertain relationships between sociodemographic variables and psychopathology in a group of Malaysian students;
- to further elucidate the relationships between psychological process variables and levels of psychopathology in a subgroup within a quarantined population.

This study will begin to demonstrate how the MCO and quarantine regulations are impacting different sociodemographic groups, the degree of psychological distress, and how these differences can be accounted for by psychological process variables. This will help inform current responses, both policy and clinical, and determine directions for future interventions for those significantly impacted by the COVID-19 pandemic.

## 2. Materials and Methods

Ethics approval was obtained from University Malaysia Sabah Medical Ethics Review Committee prior to commencement of this study.

### 2.1. Participants

A total of 515 respondents participated in this study. Participants were students enrolled in a public university on Borneo Island who were subject to the nationwide Movement Control Order as detailed previously. Snowball sampling was used to recruit participants along with a university wide email request. Although there is a small number of international students in the university, all participants in this study were Malaysian citizens. All were restricted to movement within the university campus only. There are three separate campuses; a main campus containing most of the students and faculties, and two branch campuses in different cities in Borneo which have one faculty each. The main campus contains roughly 4000 students, whereas the branch campus in Labuan had 140 students who were locked down. The main campus is located in Kota Kinabalu, a major city which was heavily affected with COVID-19 cases, while the other two campuses were in smaller cities (Sandakan and Labuan) which recorded much lower COVID-19 numbers. There were students subjected to additional levels of compulsory quarantine who were enrolled in the study as well (12 students were quarantined for two weeks due to close contact with someone who tested positive for COVID-19). The students were in this mandatory quarantine when they participated in this study. The other students were allowed to generally remain in the vicinity of their hostels and nearby cafeteria and were able to have some social interaction with others if following the strict SOP. The quarantined students were isolated to their rooms, with no contact with other individuals, and food was delivered in a no-contact manner to their doorstep. The 12 quarantined students were on the same campus. These students were quarantined for 14 days, participating in this study on the seventh day of the isolation. The research team felt that this was a suitable median point for data collection which would not be overly influenced by extreme initial or departing emotions of quarantine.

### 2.2. Procedure

In stage 1 of the study, two questionnaires were distributed to the students. Questionnaires were designed in Google Forms and circulated via email to all prospective participants, with results automatically captured in Excel format via Google Forms. It was a convenience sample of students in the public university who were subject to the Movement Control Order and hence restricted to university grounds and was distributed over a period of two weeks from 1 April 2020 to 14 April 2020, in the initial full national lockdown in Malaysia. Inclusion criteria was all university students inside campus aged above 18 years old who consented for participation. Exclusion criteria were acute medical and psychiatric illness. This study was conducted primarily in the Malay language and utilized the existing Malay version of validated scales.

The Checklist for Reporting Results of Internet E-Surveys (CHERRIES) criteria were employed as per Appendix A in administering the web forms. Informed consent was obtained in the Google Forms document, and the process explained. This was a closed and voluntary survey with no incentives, and initial contact with all participants was made via the Internet. The survey was announced through electronic mailing lists on WhatsApp due to restrictions of movement. Completeness checks were performed through allowing submission only after mandatory answering of all questions, and multiple entries from the same individual were prevented via mandatory email address registration.

### 2.3. Measures

Firstly, all students provided sociodemographic information (age, gender, year of study, education level, campus of study, and whether they were quarantined or not quarantined). Secondly, students were given a DASS-21 to complete. The DASS-21 (Depression Anxiety Stress Scale is a 21-item self-report questionnaire designed to measure the severity of emotional distress (depression and anxiety, and stress) [31]. It has 7 items for each domain. Sample items include “I found it hard to wind down” and “I couldn’t seem to experience any positive feeling at all”. Participants were requested to complete the DASS-21, based on the presence of a symptoms over the previous week. Participants rated each item on a four-point Likert scale ranging from 0 (did not apply to me at all over the last week) to 3 (applied to me very much or most of the time over the past week).

The essential function of the DASS-21 was to assess the severity of the core symptoms of emotional distress (depression, anxiety and stress). Accordingly, the DASS-21 measures the severity of an individual’s symptoms in different domains after summing up total scores of each domain. Higher scores indicate greater severity. In the present study, the Malay version of the DASS-21 [32,33] was used to measure emotional distress. Appendix A shows the DASS-21 scoring template.

Stage 2 of the study was performed using data for a concurrent project, as its results were seen to be helpful in elucidating insight into the current study. This utilized additional scales on top of the main group, and ethics approval was obtained prior to the use of the concurrent study data. The Stage 2 project utilized additional scales on top of the main group, and ethics approval was obtained prior to the use of the subgroup data. This subgroup project was performed by one of the researchers in this study as well, examining relationships between psychological process variables, but due to small sample size owing to the minimal volume of students in the branch campus, it was not published.

In stage 2, on top of the sociodemographic questionnaire and DASS scores, three measurements of psychological process variables were completed: the Balanced Index of Psychological Mindedness for psychological mindedness (BIPM), the Acceptance and Action Questionnaire-II (AAQ-II) for psychological flexibility and experiential avoidance, and the Mindfulness Attention and Awareness Scale (MAAS) for state mindfulness. All scales were delivered in the Malay language version.

The BIPM is a 14-item self-report scale assessing psychological mindedness which is defined as an individual’s interest and ability in two aspects: awareness of one’s inner psychological processes (Insight) and positive attitudes about being in touch with these processes (Interest) [34,35]. Items in this scale include self-report statements like: “I am often not aware of my feelings” and “My attitude and feelings about things fascinate me”. Each item is rated on a five-point Likert scale ranging from 0 (not true) to 4 (very much true). The 7-item Interest and Insight subscales of the BIPM showed good internal consistency (Cronbach’s alpha = 0.85 and 0.76, respectively), test–retest reliability (r = 0.63 and 0.71, respectively), and construct validity (r = 0.40 with related constructs) [32]. The Malay version of the BIPM was validated with good psychometric properties using Cronbach’s alpha (α = 0.87) [22].

The AAQ-II was developed by Hayes and colleagues as a measure to assess experiential avoidance and psychological inflexibility [36]. AAQ-II includes seven items, namely “I am afraid of my feelings” and “Emotions cause problems in my life”. Higher scores reflect higher levels of psychological inflexibility. In the original study AAQ-II showed a good level of internal consistency (.84), test–retest reliability (r = 0.81, in 3 months, and r = 0.79, in 12 months), and construct validity [35]. Higher AAQ-II scores were also found to be associated with greater levels of depressive symptoms, anxiety and stress, thought suppression and psychological distress, suggesting that psychological inflexibility may function as a risk factor for mental ill-health. The psychometric study of both versions, correlated at a high level (r = 0.96), recommend the selection of the psychometrically stronger seven item version [36]. The Cronbach’s α = 0.91 in a separate Malay validation [37].

The MAAS was proposed to assess attention and awareness in everyday life. MAAS is commonly used as a mindfulness questionnaire among general populations. It was developed by Brown and Ryan for adults, in normative and clinical populations [14]. The tool consists of a 15-item self-reported single-factor scale that focused on the attention awareness component of the mindfulness construct, through statements, for example, “I find it difficult to stay focused on what’s happening in the present” and “I rush through activities without being really attentive to them”. This 15-item scale measures the frequency of mindful states in a day-to-day life, using both general and situation-specific statements. Cronbach’s α = 0.851 in the Malay validation [38].

### 2.4. Data Analysis

All data were analyzed using SPSS 25.0. Descriptive data were presented for continuous data, and frequencies for nominal or ordinal data. Skewness and kurtosis of less than ±2 were accepted as the cut off points for normality assumptions [39]. A total of 515 respondents participated. Descriptive Statistics suggest that the skewness and kurtosis was less than 2 for all continuous variables, involving the entire sample group, including total scores for depression, anxiety and stress. There was a total of 158 males (30.7%) and 355 females (68.9%). Five hundred of the students (97.1%) were distributed between first year (24.7%), second year (23.9%), third year (38.4%) and the fourth year (10.1%), and the rest were postgraduates (2.9%). There were 12 quarantined individuals (2.3%) whereas the remaining 503 respondents (97.7%) were not quarantined. Among those quarantined, six were males (50%) and another six were females (50%).

For Stage 1, correlations were calculated between depression, anxiety, and stress scores. Cronbach alphas were calculated for all scales to ensure comparable internal validity. T-tests were used to identify if there were significant differences for gender whereas Mann-Whitney U tests were used for quarantine status due to the non-normality of the sample of 12 quarantined students. ANOVA was used to identify whether there were significant differences for year of study, education level, and campus of study. Multiple regression was performed upon depression, anxiety, and stress levels, incorporating all sociodemographic variables measured as covariates.

A separate subgroup analysis was performed with 58 respondents from the Sandakan branch campus. In this subgroup analysis, it was noted that there was no statistically significant difference for depression, anxiety, and stress scores for this when campus compared to the other two campuses (Kota Kinabalu and Labuan). Skewness and kurtosis for BIPM, MAAS, and AAQ-II results in this group were all less than 1, so it was normally distributed.

Correlations were calculated between depression, anxiety, and stress scores, and all three psychological process variables as mentioned above, including Insight and Interest subscales for PM. Cronbach alphas were calculated for all scales to ensure comparable internal validity. T-tests were used to identify if there were significant differences in psychological process variables and psychopathology for gender and quarantine status. ANOVA was used to identify if there were significant differences in psychological process variables and psychopathology for year of study, education level, and campus of study. Multiple regression was performed upon depression, anxiety and stress levels, incorporating all sociodemographic variables measured as covariates.

## 3. Results

### 3.1. Analysis of Stage 1

#### 3.1.1. Descriptive Statistics for Stage 1

As noted in Table 1, 20.2% of respondents scored “moderate” and above for Depression, 25% scored “moderate” and above for Anxiety, and 14.2% scored “moderate” and above for Stress. Cronbach alpha for the three subscales of the DASS-21 showed excellent internal consistency (Depression = 0.907, Anxiety = 0.845, Stress = 0.901). Cronbach alpha was not performed for the entire DASS-21 as it consists of three subscales whose scores cannot be combined meaningfully.

#### 3.1.2. Bivariate Data Analyses

The Mann Whitney U test was performed for comparison between quarantined and non-quarantined groups as the data for the quarantined group did not fulfill normality assumptions. As Table 2 suggests, there is no significant difference between quarantined and non-quarantined groups for anxiety and stress levels. However, there is a slightly significant difference between quarantined groups and non-quarantined groups for depressive symptoms, in which quarantined groups were more depressed compared to non-quarantined group. In the quarantined group, one individual scored “severe” and three individuals scored “moderate” on depression; one individual scored “very severe” and two individuals scored “severe” on anxiety; whereas one individual scored “severe” and three scored “moderate” on stress. The others all fell within normal or mild ranges which are not clinically significant.

For gender, *t*-tests were performed. As per Table 3, there is a significant difference for all three variables, depression, anxiety and stress, in which female participants notably experienced higher level of depression (*t* = −2.917, *p* = 0.004), anxiety (*t* = −3.007, *p* = 0.003) and stress (*t* = −3.748, *p* < 0.001).

When ANOVA was performed, there was a significant difference between the three campuses for depression scores only. Upon Bonferroni correction, the significant difference remained between Labuan and Main campus scores, with Main campus scoring higher (Mean Difference = 1.355, *p* = 0.025). Otherwise upon Bonferroni correction there was no significant difference between the scores of the other campuses compared to each other.

When comparing education level, upon ANOVA testing, there was no significant difference for depression, anxiety and stress scores.

#### 3.1.3. Multiple Regression Analyses

Subsequently multiple regressions were performed to estimate the effect of age, quarantine status, gender, and campus on depression, anxiety and stress. As per Table 4, gender (β = 0.127, t = 2.874, *p* = 0.004) and quarantine status (β = 0.103, t = 2.335, *p* = 0.020) had significant effects when regressed upon depression scores. However, year of study and campus did not have any significant effect upon regression.

Only gender (β = 0.124, t = 2.813, *p* = 0.005) had significant effects, when regressed, upon anxiety scores as per Table 5. Quarantine status, year of study and campus did not have any significant effect upon regression.

Gender (β = 0.160, t = 3.643, *p* < 0.001) had significant effects, when regressed, upon stress scores as per Table 6. However, year of study and campus did not have any significant effect upon regression.

### 3.2. Analysis of Stage 2

#### 3.2.1. Bivariate Analyses

Mann-Whitney U tests demonstrated no significant difference in Stage 2 between gender for all the psychopathology measured (depression, anxiety and stress scores) and the psychological process variables (mindfulness, psychological mindedness, and psychological flexibility). There were 38 females (65.5%) and 20 males (34.5%) in Stage 2. ANOVA demonstrated no significant difference in this subgroup between year of study for either depression, anxiety and stress scores or mindfulness, psychological mindedness, and psychological flexibility levels.

#### 3.2.2. Correlations between Psychological Process Variables and Psychopathology

As per Table 7 below, depression, anxiety and stress scores are significantly and strongly correlated, similarly to the main study. The psychological process variables themselves are considerably correlated. Psychological mindedness (PM is inversely correlated to psychological flexibility (PF) (r = −0.423, *p* = 0.001), whereas neither psychological mindedness nor psychological flexibility are correlated to mindfulness.

Looking at the correlations between psychological process variables and scores for psychopathology, depression scores are correlated inversely with psychological mindedness (r = −0.399, *p* < 0.001), in particular the “Insight” subscale (r = −0.523, *p* < 0.001); however, depression is not significantly correlated with the “Interest” subscale. Depression is not correlated with mindfulness scores, but it is inversely correlated with psychological flexibility (r = −0.469, *p* < 0.001).

Anxiety scores are correlated inversely with psychological mindedness (r = −0.429, *p* < 0.001), in particular the “Insight” subscale (r = −0.687, *p* < 0.001); however, anxiety is not significantly correlated with the “Interest” subscale. Anxiety is not correlated with mindfulness scores, but it is significantly correlated with psychological flexibility (r = 0.484, *p* < 0.001).

Stress scores are correlated inversely with psychological mindedness (r = −0.384, *p* < 0.001), in particular the “Insight” subscale (r = −0.612, *p* < 0.001); however, anxiety is not significantly correlated with the “Interest” subscale. Stress is not correlated with mindfulness scores, but it is significantly correlated with psychological flexibility (r = 0.450, *p* < 0.001).

## 4. Discussion

These findings are crucial in determining the level of psychological distress during the Movement Control Order (MCO) in Malaysia. Thus far, there has been scant literature quantifying this psychological distress in the early surge of the pandemic, with mostly opinion pieces, case reports, and general overviews prevailing [40,41,42]. Parallel literature from other countries indicates that there are higher levels of panic disorder, anxiety, and depression [1,43,44]. Tyrer has also mentioned a specific type of health anxiety that may manifest in this pandemic, COVID-19 health anxiety, in which the symptoms may overlap with the real symptoms of COVID-19 and cause diagnostic dilemma and long-term consequences, especially as the end of this pandemic is unknown [45].

This current study is novel in that it captures data about interactions between mindfulness, psychological flexibility, and psychological mindedness and three different indices of distress, namely depression, anxiety and stress. It considers these interactions uniquely from the vantage point of an early period of the pandemic. At that time there was uncertainty and fear throughout society, and it was impossible to gauge the direction the pandemic and consequent lockdown measures would take [46,47]. It captures the psychological zeitgeist of a watershed movement in history and can be instructive in the event subsequent pandemics occur. This study is also instructive in that it adds to the evidence that we already have about undergraduate students in lockdown, namely that they suffered increasing severity of anxiety and depressive symptoms [48], manifested in poorer sleep quality, poorer academic performance and increased suicidal thoughts in the university populations [49,50,51,52,53,54,55,56,57,58].

In this study, 20.2% of respondents scored “moderate” and above for Depression, 25% scored “moderate” and above for Anxiety, and 14.2% scored “moderate” and above for Stress. This compares with a large-scale study from China in the recent COVID19 pandemic, indicating that 16.5% reported moderate to severe depressive symptoms; 28.8% reported moderate to severe anxiety symptoms; and 8.1% reported moderate to severe stress symptoms [43]. Longitudinally, however, there were no significant changes in stress, anxiety and depression levels after one month in a follow-up study [44]. In a similar study conducted in Austria, among the general public, 26.5% were reported to have moderate (13.3%) to severe (13.2%) depression, 20.3% had moderate (8.9%) to severe (11.4%) anxiety and 21.2% suffered from moderate (10.5%) or severe stress (10.7%) [59]. One Polish study, which had a more comparable sample to this current group of university students, reported a prevalence of 43.4% for depression, 27.3% for anxiety and 41.0% for stress [60]. Anxiety was the most commonly reported psychopathology in the China study, comparable to our study. In contrast, the Austrian and Polish studies reported depression as the most common psychopathology. However, the Malaysian study was conducted with a group of undergraduates, which is a more homogenous group compared to snowball sampling performed in the general public. Compared to the similar Polish study carried out among university students, our study reported less psychological impact in terms of DASS-21 score. Corroborating the results with other cross-sectional studies in exploring the psychopathology levels during this pandemic, the female gender was noted to have higher levels of depression, anxiety and stress as compared to the male gender. The literature has discussed gender differences in mood disorders, including anxiety disorders and stress-related disorders, which are more prevalent in females. Similar trends were reported during previous pandemics [61,62,63,64]. During the Severe Acute Respiratory Syndrome (SARS) epidemic in early 2000, to Ebola and the Middle East Respiratory Syndrome (MERS), in which quarantine was one of the essential public health measures to break the transmission, higher levels of psychopathology were also reported, especially in anxiety and stress-related disorders [65,66,67]. These trends could also be observed during the COVID-19 pandemic, corroborating the finding that quarantine, albeit necessary, may precipitate and perpetuate psychological distress for affected individuals.

Findings indicate there is a significant difference between quarantined groups and non-quarantined groups for depressive symptoms. This is concurrent with, and adds statistical support to, a recent rapid review of the evidence suggesting there are clear differences between these two groups [68]. The other key finding is that gender predicted changes in depression, anxiety, and stress across the board. Hence, quarantined groups and female groups need increased support and attention as they can be at higher risk of developing psychopathology.

The subgroup analysis aimed to further explore potential psychological process variables that underlie the depressive, anxiety and stress symptoms. Psychological mindedness, psychological flexibility, and state mindfulness were predicted to covary from the literature [35,69,70]. This study however demonstrated the converse; mindfulness did not demonstrate statistically significant correlations with the other two psychological process variables. Another surprising correlation was that psychological mindedness and psychological flexibility were inversely correlated. One possibility is that the MAAS measures state, rather than trait, mindfulness [14]. State mindfulness refers to short-term fluctuations in mindfulness in response to a current stressor, whereas trait mindfulness is a long-term phenomenon of mindfulness that is less easily influenced by current stressors. Under the high level of uncertainty and constant anxiety of living under the MCO, it may be hard for individuals to have a short-term level of improved mindfulness. In contrast, PM and PF are more “trait” qualities, which are more enduring and persistent [34,36]. Hence, individuals with higher PM and PF may cope better in the MCO and have less psychological distress.

The findings that psychological mindedness is the only constant predictor of all three psychopathology—depression, anxiety, and stress—corroborate extant literature [11,35]. This suggests that, instead of merely providing physical needs, psychological interventions or programs designed to increase one’s insight and interest in one’s owns feelings and thoughts could be offered as they have statistically significant effects on depressive, anxiety, and stress symptoms [22]. Overall, this study adds some theoretical scaffolding to a very practical issue, namely that an MCO would understandably make individuals more depressed, stressed and anxious [69,71]. This study considers a second, deeper layer of psychological mechanisms to identify practical clinical steps that can be taken. Realizing the potential of an improved psychological mindedness construct, it is crucial we train people to perform brief psychological interventions that have been shown to improve psychological mindedness [72]. Suggestions that have been employed by UMS in this direction include small focus groups online, randomized psychological first aid calls to students and frontline workers, and an online teleconsultation portal to perform brief psychological interventions. As the pandemic enters its second year, various evidence-based strategies that have been employed in this sample group include online mindfulness-based interventions targeting psychological mindedness [73], the setting up of Dialectical Behavior Therapy skills training groups as part of an overall intervention for students with higher levels of psychopathology, and transitioning of offline activities such as quiz competitions and international debate competitions to an online format successfully to reduce isolation and increase connectedness and a sense of belonging [46].

This study no doubt exhibits a few limitations. It is crucial that we examine the results of this study with caution, especially as many of the hypotheses are constructed necessarily using pre-pandemic data, and also due to multiple restrictions on face to face data collection which prevent us from fully ascertaining the fidelity of the data collection process. First; it is a cross sectional study so only association through correlation analysis rather than causation can be established. Second; all data collection was done online due to social distancing requirements so there is no opportunity to filter out data incongruous to respondents. Third; due to logistical difficulties, only a small group was able to participate in the second subgroup analysis. However, from the *t*-tests performed, there is no statistical difference between the main group and the second group, making the data comparable in composition. We did not examine disciplinary differences, which may have yielded significant results for particular disciplines, such as medical students. Fourth; only one scale was used to measure depression, anxiety and stress. However, the scale has been extensively validated in the Malaysian population. Fifth; there were two unavoidable sample size issues: only 12 of the 512 main campus respondents were quarantined, reducing the power of statistical analysis. This was a factor that we were unable to control as that was the real time number of quarantined students during the time period. The subgroup analysis was conducted with a group that was also small in size, limiting generalizability. Lastly, due to the presence of the MCO, it may have artificially inflated the psychopathology scores especially for anxiety and stress. However, the anxiety and stress scores are still below the community prevalence of generalized anxiety disorder and panic disorder [74].

There are multiple future implications for this research. There are particular factors, such as quarantine, that result in individuals exhibiting significantly higher levels of psychopathology, hence there must be provision of telehealth or phone-based psychological first aid consultations to prevent mental health treatment gaps occurring in those who are forcibly secluded from contact. At the same time, this study underscores the higher presence of psychopathology in females, which needs to be addressed proactively via primary prevention and reaching out to female populations rather than reactive referral pathways which can increase morbidity.

## 5. Conclusions

In conclusion, the COVID-19 pandemic has understandably resulted in significant increases in mental health concerns around the world. However, this study goes one step further by assessing the relationship between mental health concerns and various psychological process variables. This study, importantly, is novel in that it captures the psychological processes and interactions with psychopathology at a period in time where the world was still fraught with high levels of uncertainty and fear regarding COVID-19. Psychological mindedness has in this study again emerged as a promising factor that appears to in some ways elucidate how depression, anxiety and stress form. Various psychological interventions, ranging from simple psychological first aid and counselling, to CBT and mindfulness, can help increase psychological mindedness [72]. Ultra-brief psychological interventions have been found to help in these situations [75], and to alleviate the behaviors associated with fear, anxiety and stigma [76]. The clinical and operational implications of this research suggest widening access to such psychological interventions. This requires creativity in an age of social distancing and pandemic concerns. However, many of our planet’s greatest leaps forward in medicine, technology, and science have come in periods of great privations and stress, for instance during the World Wars, and it is hoped that the mental health fraternity will have the resources to rise to the challenge and increase its outreach dramatically. This can be made available using online and telephone services, enabling quarantined and isolated individuals access to essential supports and strategies to manage the impact of this on their mental health. The responsibilities of mental health fraternity are definitely emphasized by this pandemic, which could be the silver lining from this crisis, as mental health or psychiatry is brought to the attention of healthcare administrators as well as the public. They are a necessity and the key to supporting the mental health of existing, as well as potential, patients resulting from this pandemic, the general public, healthcare colleagues and the whole healthcare system, through prevention, diagnosis and management with the incorporation of technologies in this era [77,78,79,80].

## Figures and Tables

**Table 1:** Distribution of Depression, Anxiety and Stress Scores by Percentage.

Level of Scoring	Depression, n ^†^ (%)	Anxiety, n ^†^ (%)	Stress, n ^†^ (%)
Normal	276 (53.6)	289 (56.2)	381 (74.0)
Mild	135 (26.2)	97 (18.8)	61 (11.8)
Moderate	54 (10.5)	67 (13.0)	50 (9.7)
Severe	32 (6.2)	28 (5.4)	15 (2.9)
Extremely Severe	18 (3.5)	34 (6.6)	8 (1.6)

^†^ n = 515.

**Table 2:** Difference between quarantined and non-quarantined status for DASS categories.

DASS Categories	Mean (Quarantine)	Mean (Not Quarantined)	Mann Whitney U Test	
			U	Sig. (2-Tailed) *
Depression	7.75	4.96	4154.000	0.025
Anxiety	5.75	4.44	3468.000	0.375
Stress	7.50	5.67	3827.000	0.110

* Significant at the 0.05 level (two-tailed test).

**Table 3:** Difference between female and male for DASS categories.

DASS Categories	Mean (Female)	Mean (Male)	*t*	df	Sig. (2-Tailed) *	95% Confidence Interval of the Difference	
						Lower	Upper
Depression	5.38	4.21	−2.917	511	0.004	−1.965	−0.383
Anxiety	4.77	3.74	−3.007	511	0.003	−1.710	−0.358
Stress	6.15	4.69	−3.748	511	0.000	−2.224	−0.694

* Significant at the 0.05 level (two-tailed test).

**Table 4:** Multiple regression with depression as dependent variable.

ANOVA ^a^						
Model		Sum of Squares	df	Mean Square	F	Sig.
1	Regression	273.488	4	68.372	3.889	0.004 ^b^
	Residual	8931.276	508	17.581		
	Total	9204.764	512			
**Coefficients ^c^**						
**Model**		**Unstandardized Coefficients**		**Standardized** **Coefficients**	**t**	**Sig. ^†^**
		**B**	**Std. Error**	**Beta**		
1	(Constant)	0.677	1.656		0.409	0.683
	Gender	1.162	0.404	0.127	2.874	0.004
	Quarantine status	2.877	1.232	0.103	2.335	0.020
	Year of study	−0.040	0.177	−0.010	−0.226	0.821
	Campus	−0.226	0.207	−0.048	−1.089	0.277

^a^ Dependent Variable: Depression; ^b^ Predictors: (Constant). Gender, quarantine status, year of study, campus. ^c^ Dependent Variable: Depression. ^†^ Significant at the 0.05 level.

**Table 5:** Multiple regression with anxiety as dependent variable.

ANOVA ^a^						
Model		Sum of Squares	df	Mean Square	F	Sig.
1	Regression	184.897	4	46.224	3.589	0.007 ^b^
	Residual	6542.366	508	12.879		
	Total	6727.263	512			
**Coefficients ^c^**						
**Model**		**Unstandardized Coefficients**		**Standardized** **Coefficients**	**t**	**Sig. ^†^**
		**B**	**Std. Error**	**Beta**		
1	(Constant)	2.420	1.417		1.707	0.088
	Gender	0.974	0.346	0.124	2.813	0.005
	Quarantine status	1.257	1.055	0.052	1.192	0.234
	Year of study	−0.237	0.152	−0.069	−1.560	0.119
	Campus	−0.156	0.177	−0.039	−0.878	0.381

^a^ Dependent Variable: Anxiety. ^b^ Predictors: (Constant). Gender, quarantine status, year of study, campus. ^c^ Dependent Variable: Anxiety. ^†^ Significant at the 0.05 level.

**Table 6:** Multiple regression with stress as dependent variable.

ANOVA ^a^						
Model		Sum of Squares	df	Mean Square	F	Sig.
1	Regression	300.617	4	75.154	4.543	0.001 ^b^
	Residual	8403.153	508	16.542		
	Total	8703.770	512			
**Coefficients ^c^**						
**Model**		**Unstandardized Coefficients**		**Standardized** **Coefficients**	**t**	**Sig. ^†^**
		**B**	**Std. Error**	**Beta**		
1	(Constant)	1.973	1.606		1.228	0.220
	Gender	1.429	0.392	0.160	3.643	0.000
	Quarantine status	1.901	1.195	0.070	1.591	0.112
	Year of study	−0.127	0.172	−0.033	−0.742	0.459
	Campus	−0.158	0.201	−0.034	−0.784	0.434

^a^ Dependent Variable: Stress. ^b^ Predictors: (Constant). Gender, quarantine status, year of study, campus. ^c^ Dependent Variable: Stress. ^†^ Significant at the 0.05 level.

**Table 7:** Correlations between psychological process variables and psychopathology in subgroup analysis (n = 58).

	PM ^a^	PM—Insight ^a^	PM—Interest ^a^	State Mindfulness ^b^	PF ^c^	Depression	Anxiety
PM—Insight	0.669 **	-	-	-	-	-	-
PM—Interest	0.514 **	−0.293 *	-	-	-	-	-
State mindfulness	0.054	0.082	−0.025	-	-	-	-
PF	−0.423 **	−0.712 **	0.277 *	−0.077	-	-	-
Depression	−0.399 **	−0.523 **	0.091	−0.257	−0.469 **	-	-
Anxiety	−0.429 **	−0.687 **	0.240	−0.202	0.484 **	0.721 **	-
Stress	−0.384 **	−0.612 **	0.213	−0.259	0.450 **	0.845 **	0.383 **

* Correlation is significant at the 0.05 level (two-tailed); ** Correlation is significant at the 0.001 level (two-tailed). ^a^ PM, PM—Insight, PM—Interest: measured through Balanced Index of Psychological Mindedness. ^b^ State mindfulness: measured through Mindfulness Attention and Awareness Scale. ^c^ PF: measured through Acceptance and Action Questionnaire-II.

## Data Availability

The data presented in this study are available on request from the corresponding author. The data are not publicly available due to privacy issues.

## Abbreviation

MCO	Movement Control Order
PM	Psychological Mindedness
PF	Psychological Flexibility
BIPM	Balanced Index of Psychological Mindedness
MAAS	Mindful Attention Awareness Scale
AAQ-II	Acceptance and Action Questionnaire-II
UMS	Universiti Malaysia Sabah
CBT	Cognitive Behavioral Therapy

## Appendix A

Checklist for Reporting Results of Internet E-Surveys (CHERRIES).

**Checklist Item**	**Explanation**
Describe survey design	The target population is university undergraduates and the sample frame is students who are locked down in the university. The sample is a convenience sample.
IRB approval	The study has been approved by an IRB as mentioned in the article.
Informed consent	Informed consent was obtained in the Google Forms document, and the process explained.
Data protection	The Google Forms document was password protected with only the researchers able to access its data.
Development and testing	The usability and technical functionality of the electronic questionnaire had been tested before fielding the questionnaire. The survey instruments had been tested before in paper format with good statistical properties.
Open survey versus closed survey	This was an open survey.
Contact mode	The initial contact with the potential participants was made on the Internet.
Advertising the survey	The survey was announced through electronic mailing lists on WhatsApp groups which were participated in by all students in the university lockdown, due to restrictions of movement.
Web/E-mail	This was a survey recruited through a link in a WhatsApp group.
Context	The WhatsApp group with the survey was one that was
Mandatory/voluntary	It was a voluntary survey.
Incentives	No incentives were offered.
Time/Date	The data was collected over a month from 1–30 April 2020
Randomization of items or questionnaires	Items were randomized to prevent bias.
Adaptive questioning	Adaptive questioning was not used as there was insufficient time to validate a truncated version of the questionnaire in time.
Number of Items	Each questionnaire was on a separate page, so the maximum number of questions on a page was 21.
Number of screens (pages)	It was distributed over 4 pages.
Completeness check	Completeness checks were performed through allowing submission only after mandatory answering of all questions, and multiple entries from the same individual were prevented via mandatory email address registration.
Review step	Respondents were not able to review and change their answers due to the Google Forms format.
Unique site visitor	We determined unique site visitors based on email addresses furnished, and all non-unique email addresses were parsed out.
View rate (Ratio of unique survey visitors/unique site visitors)	We were not able to calculate a view rate as we did not have a website to visit, just a Google Form link.
Participation rate (Ratio of unique visitors who agreed to participate/unique first survey page visitors)	Again, as this was done through Google Forms, which does not tabulate the number of visitors who agreed to participate on the first page, but only tabulates form completion, we were unable to assess this.
Completion rate (Ratio of users who finished the survey/users who agreed to participate)	Similarly, as this was done through Google Forms, which does not tabulate the number of visitors who agreed to participate on the first page, but only tabulates form completion, we were unable to assess this.
Cookies used	Cookies were not used to assign a unique user identifier to each client computer.
IP check	The IP address of the client computer was not used to identify potential duplicate entries from the same user.
Log file analysis	No other techniques to analyze the log file for identification of multiple entries were used.
Registration	Not relevant
Handling of incomplete questionnaires	We were only able to analyse completed questionnaires as Google Forms did not store incomplete questionnaires.
Questionnaires submitted with an atypical timestamp	We were not able to measure starting and ending timestamps in Google Forms so were unable to assess for atypical timestamps
Statistical correction	Methods such as weighting of items or propensity scores were not used to adjust for the non-representative sample.

## References

[1] Qiu J., Shen B., Zhao M., Wang Z., Xie B., Xu Y. (2020). A nationwide survey of psychological distress among Chinese people in the COVID-19 epidemic: Implications and policy recommendations. Gen. Psychiatry.

[2] Marazziti D., Stahl S.M. (2020). The relevance of COVID-19 pandemic to psychiatry. World Psychiatry.

[3] Ghebreyesus T.A. (2020). Addressing mental health needs: An integral part of COVID-19 response. World Psychiatry.

[4] Pedrelli P., Nyer M., Yeung A., Zulauf C., Wilens T. (2015). College students: Mental health problems and treatment considerations. Acad. Psychiatry.

[5] Wyatt T., Oswalt S.B. (2013). Comparing mental health issues among undergraduate and graduate students. Am. J. Health Educ..

[6] Mukhsam M.H., Jeffree M.S., Pang N.T.P., Rahim S.S.S.A., Omar A., Abdullah M.S., Lukman K.A., Giloi N., Salvaraji L., Karim M.R.A. (2020). A University-Wide Preparedness Effort in the Alert Phase of COVID-19 Incorporating Community Mental Health and Task-Shifting Strategies: Experience from a Bornean Institute of Higher Learning. Am. J. Trop. Med. Hyg..

[7] Salvaraji L., Sharizman Syed Abdul Rahim S., Saffree Jeffree M., Omar A., Tze Ping Pang N., Ahmedy F., Hayati F., Tat Yeap B., Giloi N., Saupin S. (2020). The importance of high index of suspicion and immediate containment of suspected COVID-19 cases in institute of higher education Sabah, Malaysia Borneo. Malays. J. Public Health Med..

[8] Musiun A., Lukman K.A., Jeffree M.S., Robinson F., Hassan M.R., Ghazi H., Al-Abed A.-A.A.A., Tha N.O., Swe, Shamsudin S.B. (2019). Prevalence of stress and its associated factors among medical students in Sabah, Malaysia Borneo. Malays. J. Public Health Med..

[9] Pang N., Imon G.N., Johoniki E., Kassim M.M., Omar A., Rahim S.S.A., Hayati F., Jeffree M., Ng J. (2021). Fear of COVID-19 and COVID-19 Stress and Association with Sociodemographic and Psychological Process Factors in Cases under Surveillance in a Frontline Worker Population in Borneo. Int. J. Environ. Res. Public Health.

[10] Grant A.M. (2001). Rethinking Psychological Mindedness: Metacognition, Self-reflection, and Insight. Behav. Chang..

[11] Trudeau K.J., Reich R. (1995). Correlates of psychological mindedness. Pers. Individ. Differ..

[12] Nyklíček I., Majoor D., Schalken P.A.A.M. (2010). Psychological mindedness and symptom reduction after psychotherapy in a heterogeneous psychiatric sample. Compr. Psychiatry.

[13] Zvolensky M.J., Solomon S.E., McLeish A.C., Cassidy D., Bernstein A., Bowman C.J., Yartz A.R. (2006). Incremental validity of mindfulness-based attention in relation to the concurrent prediction of anxiety and depressive symptomatology and perceptions of health. Cogn Behav Ther..

[14] MacKillop J., Anderson E.J. (2007). Further psychometric validation of the mindful attention awareness scale (MAAS). J. Psychopathol. Behav. Assess..

[15] Hayes S.C., Strosahl K.D., Wilson K.G. (2011). Acceptance and Commitment Therapy: The Process and Practice of Mindful Change.

[16] Kashdan T.B., Rottenberg J. (2010). Psychological flexibility as a fundamental aspect of health. Clin. Psychol. Rev..

[17] Carvalho S., Martins C.P., Almeida H.S., Silva F. (2017). The Evolution of Cognitive Behavioural Therapy—The Third Generation and Its Effectiveness. Eur. Psychiatry.

[18] Dimidjian S., Arch J.J., Schneider R.L., Desormeau P., Felder J.N., Segal Z.V. (2016). Considering Meta-Analysis, Meaning, and Metaphor: A Systematic Review and Critical Examination of “Third Wave” Cognitive and Behavioral Therapies. Behav. Ther..

[19] Silberstein L.R., Tirch D., Leahy R.L., McGinn L. (2012). Mindfulness, psychological flexibility and emotional schemas. Int. J. Cogn. Ther..

[20] Appelbaum S.A. (1973). Psychological mindedness: Word, concept and essence. Int. J. Psychoanal..

[21] Bond F.W., Flaxman P.E., van Veldhoven M.J.P.M., Biron M., Houdmont J., Leka S. (2010). The Impact of Psychological Flexibility and Acceptance and Commitment Therapy (act) on Health and Productivity at Work. Contemporary Occupational Health Psychology: Global Perspectives on Research and Practice.

[22] Mohd Amiruddin M.K., Nicholas Pang T.P., Wendy D.S., Wen L.T., Abbylen M.E. (2021). Validation of Bahasa Malaysia Version of Psychological Mindedness in a University Population. IIUM Med. J. Malays..

[23] Giromini L., Brusadelli E., Di Noto B., Grasso R., Lang M. (2015). Measuring psychological mindedness: Validity, reliability, and relationship with psychopathology of an Italian version of the Balanced Index of Psychological Mindedness. Psychoanal. Psychother..

[24] Conversano C., Di Giuseppe M., Miccoli M., Ciacchini R., Gemignani A., Orrù G. (2020). Mindfulness, age and gender as protective factors against psychological distress during COVID-19 pandemic. Front. Psychol..

[25] Reiter K., Ventura J., Lovell D., Augustine D., Barragan M., Blair T., Chesnut K., Dashtgard P., Gonzalez G., Pifer N. (2020). Psychological Distress in Solitary Confinement: Symptoms, Severity, and Prevalence in the United States, 2017–2018. Am. J. Public Health..

[26] Haney C. (2003). Mental health issues in long-term solitary and “supermax” confinement. Crime Delinq..

[27] Cloyes K.G., Lovell D., Allen D.G., Rhodes L.A. (2006). Assessment of psychosocial impairment in a supermaximum security unit sample. Crim. Justice Behav..

[28] Pang N.T.P., Kamu A., Hambali N.L.B., Mun H.C., Kassim M.A., Mohamed N.H., Ayu F., Rahim S.S.S.A., Omar A., Jeffree M.S. (2020). Malay Version of the Fear of COVID-19 Scale: Validity and Reliability. Int. J. Ment. Health Addict..

[29] Kassim M.A.M., Ayu F., Kamu A., Pang N.T.P., Ho C.M., Algristian H., Sahri M., Hambali N.L.B., Omar A. (2020). Indonesian Version of the Fear of COVID-19 Scale: Validity and Reliability. Borneo Epidemiol. J..

[30] Kassim M.A.M., Pang N.T.P., Mohamed N.H., Kamu A., Ho C.M., Ayu F., Rahim S.S.S.A., Omar A., Jeffree M.S. (2021). Relationship Between Fear of COVID-19, Psychopathology and Sociodemographic Variables in Malaysian Population. Int. J. Ment. Health Addict..

[31] Lovibond P.F., Lovibond S.H. (1995). The structure of negative emotional states: Comparison of the Depression Anxiety Stress Scales (DASS) with the Beck Depression and Anxiety Inventories. Behav. Res. Ther..

[32] Ramli M., Rosnani S., Aidil Faszrul A.R. (2012). Psychometric profile of malaysian version of the Depressive, Anxiety and Stress Scale 42-item (DASS-42). Malays. J. Psychiatry.

[33] Musa R., Fadzil M.A., Zain Z. (2007). Translation, validation and psychometric properties of Bahasa Malaysia version of the Depression Anxiety and Stress Scales (DASS). ASEAN J. Psychiatry.

[34] Nykliček I., Denollet J. (2009). Development and evaluation of the Balanced Index of Psychological Mindedness (BIPM). Psychol. Assess..

[35] Nykliček I., Poot J.C., van Opstal J. (2010). Psychological mindedness in relation to personality and coping in a sample of young adult psychiatric patients. J. Clin. Psychol..

[36] Bond F.W., Hayes S., Baer R.A., Carpenter K.M., Guenole N., Orcutt H., Waltz T., Zettle R.D. (2011). Preliminary psychometric properties of the Acceptance and Action Questionnaire--II: A revised measure of psychological inflexibility and experiential avoidance. Behav. Ther..

[37] Shari N.I., Zainal N.Z., Guan N.C., Ahmad Sabki Z., Yahaya N.A. (2019). Psychometric properties of the acceptance and action questionnaire (AAQ II) Malay version in cancer patients. PLoS ONE.

[38] Zainal N.Z., Nor-Aziyan Y., Subramaniam P. (2015). Psychometric properties of the Malay-translated Mindfulness, Attention and Awareness Scale (MAAS) in a group of nursing students in Malaysia. Malays. J. Psychiatry.

[39] Kline R.B., Santor D.A. (1999). Principles & practice of structural equation modelling. Can. Psychol..

[40] Zulkifli N.A., Sivapatham S., Guan N.C. (2020). Brief Psychotic Disorder in Relation to Coronavirus, COVID-19 Outbreaks: A Case Report. Malays. J. Psychiatry..

[41] Abdullah J.M., Ismail W.F.N.W.A.N., Mohamad I., Ab Razak A., Harun A., Musa K.I., Lee Y.Y. (2020). A Critical Appraisal of COVID-19 in Malaysia and Beyond. Malays. J. Med Sci..

[42] Guan N.C., Sue-Yin L., Shiang L.X. (2020). Panicdemic in Light of Pandemic. Malays. J. Psychiatry.

[43] Wang C., Pan R., Wan X., Tan Y., Xu L., Ho C.S., Ho R.C. (2020). Immediate psychological responses and associated factors during the initial stage of the 2019 coronavirus disease (COVID-19) epidemic among the general population in China. Int. J. Environ. Res. Public Health.

[44] Wang C., Pan R., Wan X., Tan Y., Xu L., McIntyre R.S., Choo F.N., Tran B., Ho R., Sharma V. (2020). A longitudinal study on the mental health of general population during the COVID-19 epidemic in China. Brain Behav. Immun..

[45] Tyrer P. (2020). COVID-19 health anxiety. World Psychiatry.

[46] Pang N.T.P., Kamu A., Kassim M.A.M., Ho C.M. (2021). Monitoring the impact of Movement Control Order (MCO) in flattening the cummulative daily cases curve of Covid-19 in Malaysia: A generalized logistic growth modeling approach. Infect. Dis. Model..

[47] Pang N.T.P., Kamu A., Mohd Kassim M.A., Chong Mun H. (2021). Analyses of the Effectiveness of Movement Control Order (MCO) in Reducing the COVID-19 Confirmed Cases in Malaysia. J. Health Transl. Med..

[48] Kassim M.A.M., Pang N.T.P., Kamu A., Arslan G., Mohamed N.H., Zainudin S.P., Ayu F., Ho C.M. (2021). Psychometric Properties of the Coronavirus Stress Measure with Malaysian Young Adults: Association with Psychological Inflexibility and Psychological Distress. Int. J. Ment. Health Addict..

[49] Wang C., Zhao H. (2020). The Impact of COVID-19 on Anxiety in Chinese University Students. Front. Psychol..

[50] Mudenda S., Zulu A., Phiri M.N., Ngazimbi M., Mufwambi W., Kasanga M., Banda M. (2020). Impact of Coronavirus Disease 2019 (COVID-19) on College and University Students: A Global Health and Education Problem. Aquademia.

[51] Wathelet M., Duhem S., Vaiva G., Baubet T., Habran E., Veerapa E., Debien C., Molenda S., Horn M., Grandgenèvre P. (2020). Factors Associated with Mental Health Disorders Among University Students in France Confined During the COVID-19 Pandemic. JAMA Netw. Open.

[52] Van Nguyen D., Pham G.H., Nguyen D.N. (2020). Impact of the Covid-19 pandemic on perceptions and behaviors of university students in Vietnam. Data Brief.

[53] Odriozola-González P., Planchuelo-Gómez Á., Irurtia M.J., de Luis-García R. (2020). Psychological effects of the COVID-19 outbreak and lockdown among students and workers of a Spanish university. Psychiatry Res..

[54] Padrón I., Fraga I., Vieitez L., Montes C., Romero E. (2021). A Study on the Psychological Wound of COVID-19 in University Students. Front. Psychol..

[55] Akhtarul Islam M., Barna S.D., Raihan H., Nafiul Alam Khan M., Tanvir Hossain M. (2020). Depression and anxiety among university students during the COVID-19 pandemic in Bangladesh: A web-based cross-sectional survey. PLoS ONE.

[56] Browning M.H.E.M., Larson L.R., Sharaievska I., Rigolon A., McAnirlin O., Mullenbach L., Cloutier S., Vu T.M., Thomsen J., Reigner N. (2021). Psychological impacts from COVID-19 among university students: Risk factors across seven states in the United States. PLoS ONE.

[57] Traunmüller C., Stefitz R., Gaisbachgrabner K., Schwerdtfeger A. (2020). Psychological correlates of COVID-19 pandemic in the Austrian population. BMC Public Health.

[58] Juchnowicz D., Baj J., Forma A., Karakuła K., Sitarz E., Bogucki J., Karakula-Juchnowicz H. (2021). The Outbreak of SARS-CoV-2 Pandemic and the Well-Being of Polish Students: The Risk Factors of the Emotional Distress during COVID-19 Lockdown. J. Clin. Med..

[59] Fernandes A.J. (2020). Impact of COVID-19: University Students’ Perspective. Int. J. Nutr. Pharmacol. Neurol. Diseases..

[60] Mohd Kassim M.A., Pang N., James S. (2020). COVID-19 Pandemic—A Review and Assessing Higher Education Institution Undergraduate Student’s Mental Health. Borneo Epidemiol. J..

[61] Seney M.L., Sibille E. (2014). Sex differences in mood disorders: Perspectives from humans and rodent models. Biol. Sex Differ..

[62] Albert P.R. (2015). Why is depression more prevalent in women?. J. Psychiatry Neurosci. JPN.

[63] Bangasser D.A., Valentino R.J. (2014). Sex differences in stress-related psychiatric disorders: Neurobiological perspectives. Front. Neuroendocr..

[64] McLean C.P., Asnaani A., Litz B.T., Hofmann S.G. (2011). Gender differences in anxiety disorders: Prevalence, course of illness, comorbidity and burden of illness. J. Psychiatr. Res..

[65] Hawryluck L., Gold W.L., Robinson S., Pogorski S., Galea S., Styra R. (2004). SARS control and psychological effects of quarantine, Toronto, Canada. Emerg. Infect. Dis..

[66] Reynolds D.L., Garay J.R., Deamond S.L., Moran M.K., Gold W., Styra R. (2008). Understanding, compliance and psychological impact of the SARS quarantine experience. Epidemiol. Infect..

[67] Lee S.M., Kang W.S., Cho A.-R., Kim T., Park J.K. (2018). Psychological impact of the 2015 MERS outbreak on hospital workers and quarantined hemodialysis patients. Compr. Psychiatry..

[68] Brooks S.K., Webster R.K., Smith L.E., Woodland L., Wessely S., Greenberg N., Rubin G.J. (2020). The psychological impact of quarantine and how to reduce it: Rapid review of the evidence. Lancet.

[69] Beitel M., Ferrer E., Cecero J.J. (2004). Psychological mindedness and cognitive style. J. Clin. Psychol..

[70] Germer C., Siegel R.D., Fulton P.R. (2016). Mindfulness and Psychotherapy.

[71] Ho C.S.H., Chee C.Y., Ho R.C. (2020). Mental health strategies to combat the psychological impact of COVID-19 beyond paranoia and panic. Ann. Acad. Med. Singapore.

[72] Boylan M.B. (2006). Psychological Mindedness as a Predictor of Treatment Outcome with Depressed Adolescents. Ph.D. Thesis.

[73] Pang N.T.P., Tio V.C.S., Bhupendar Singh A.S., Tseu M.W.L., Shoesmith W.D., Abd Rahim M.A., Mohd Kassim M.A. (2021). Efficacy of a single-session online ACT-based mindfulness intervention among undergraduates in lockdown amid COVID-19 pandemic. Trends Psychiatry Psychother..

[74] de Lijster J.M., Dierckx B., Utens E.M.W.J., Verhulst F.C., Zieldorff C., Dieleman G.C., Legerstee J.S. (2017). The age of onset of anxiety disorders: A meta-analysis. Can. J. Psychiatry.

[75] Pang N.T.P., Shoesmith W.D., James S., Nor Hadi N.M., Eugene Boon Yau K., Loo J.L. (2020). Ultra Brief Psychological Interventions for COVID-19 Pandemic: Introduction of a Locally-Adapted Brief Intervention for Mental Health and Psychosocial Support Service. Malays. J. Med. Sci..

[76] Koh E.B.Y., Pang N.T.P., Shoesmith W.D., James S., Nor Hadi N.M., Loo J.L. (2020). The Behaviour Changes in Response to COVID-19 Pandemic within Malaysia. Malays. J. Med. Sci..

[77] Gorwood P., Fiorillo A. (2021). One year after the COVID-19: What have we learnt, what shall we do next?. Eur. Psychiatry.

[78] Stewart D.E., Appelbaum P.S. (2020). COVID-19 and psychiatrists’ responsibilities: A WPA position paper. World Psychiatry..

[79] Unützer J., Kimmel R.J., Snowden M. (2020). Psychiatry in the age of COVID-19. World Psychiatry.

[80] Fiorillo A., Gorwood P. (2020). The consequences of the COVID-19 pandemic on mental health and implications for clinical practice. Eur. Psychiatry J. Assoc. Eur. Psychiatrists.

